# Selective Laser-Induced Etching of 3D Precision Quartz Glass Components for Microfluidic Applications—Up-Scaling of Complexity and Speed

**DOI:** 10.3390/mi8040110

**Published:** 2017-04-01

**Authors:** Jens Gottmann, Martin Hermans, Nikolai Repiev, Jürgen Ortmann

**Affiliations:** LightFab GmbH, Campus-Boulevard 79, 52074 Aachen, Germany; m.hermans@lightfab.de (M.H.); n.repiev@lightfab.de (N.R.); j.ortmann@lightfab.de (J.O.)

**Keywords:** selective laser-induced etching, 3D precision glass parts, ultrafast laser machining, quartz glass machining, subtractive 3D printing

## Abstract

By modification of glasses with ultrafast laser radiation and subsequent wet-chemical etching (here named SLE = selective laser-induced etching), precise 3D structures have been produced, especially in quartz glass (fused silica), for more than a decade. By the combination of a three-axis system to move the glass sample and a fast 3D system to move the laser focus, the SLE process is now suitable to produce more complex structures in a shorter time. Here we present investigations which enabled the new possibilities. We started with investigations of the optimum laser parameters to enable high selective laser-induced etching: surprisingly, not the shortest pulse duration is best suited for the SLE process. Secondly we investigated the scaling of the writing velocity: a faster writing speed results in higher selectivity and thus higher precision of the resulting structures, so the SLE process is now even suitable for the mass production of 3D structures. Finally we programmed a printer driver for commercial CAD software enabling the automated production of complex 3D glass parts as new examples for lab-on-a-chip applications such as nested nozzles, connectors and a cell-sorting structure.

## 1. Introduction

Pure transparent materials such as quartz glass (fused silica glass) can be processed to generate 3D structures in a subtractive 3D printing process similar to 3D lithography using the glass as positive-tone resist [1]. The exposure is executed by scanning focused ultrashort pulsed (fs or ps) laser radiation inside the glass changing its properties locally in the focal volume. The development is executed by subsequent wet-chemical etching (an acid such as HF or an alkaline such as KOH or similar materials in water). Depending on the processing conditions, the etching rate of the laser-modified material can be much larger than the untreated glass, e.g., the selectivity (the ratio of the two etching rates) can be larger than 1400:1 in quartz silica. Thus 3D structures with micrometer precision can be produced by this method called selective laser-induced etching (SLE), also known as femtosecond laser irradiation, followed by chemical etching (FLICE), femtosecond laser assisted etching (FLAE), in-volume selective laser etching (ISLE), femto-Etch^TM^ (Franklin, OH, USA) or FEMTOprint^®^ (Muzzano, Switzerland), to name a few names. The fundamentals of the process, some applications and also its combination with other ultrafast 3D writing technologies can be found in reviews such as [2,3,4] and the references therein. The state of the art is that 3D microchannels and complex movable 3D precision parts can be produced in quartz silica glass and that the SLE process is possible in plenty of transparent materials, both in crystals such as sapphire, quartz, YAG and in glasses such as borosilicate glass (e.g., borofloat^TM^ or willow^TM^), alumino silicate glasses, soda lime glass or ultra low expansion glass (ULE). However, future research is necessary to enable 3D printing of complex structures in materials different from quartz glass. In quartz glass, first-time-right 3D printing is possible today for complex 3D parts less than 7 mm in height with a precision of about 10 µm and a maximum tunnel length of 10 mm. Larger precision or longer tunnel length are feasible but often make iterations of production and measurements necessary before the part fits the precision requirements. In this paper the investigations and the developed processing strategies which enable the subtractive 3D printing of quartz glass precision parts are described. 

For future dissemination of the SLE technology, a pathway out of the 3D printing niche is needed: like most additive or subtractive 3D printing technologies, SLE is considered a technology suitable for the fabrication of prototypes and small series of 3D precision parts. In case no possibility for later mass production of a 3D structure is in view, often even the prototype is not desired, since for mass production one would have to stay with conventional manufacturing anyway, rendering all the new 3D printing magic useless for real applications. Thus, investigations to scale the SLE technology towards a production speed sufficient for mass production are done. By demonstrating the principle feasibility to scale the SLE process enabling mass production, the road is paved also for future fundamental research projects on 3D designs in transparent materials for future applications in various disciplines.

## 2. Materials and Methods

The material used in all the studies is quartz glass (fused silica) 1–7 mm in thickness with optically polished surfaces. The laser source in the LightFab 3D Printer used for the experiments is a FCPA laser (Satsuma, Amplitude Systemes, Pessac, France) providing ultrashort laser pulses with pulse duration adjusted between 300 fs and 1 ps, repetition rate varied between 250 kHz and −14 MHz, average power up to 10 W at 1 MHz and a wavelength of 1030 nm. The laser radiation is focused inside the glass workpiece using 20× plan field microscope objective lenses with focal length of 8, 9 or 10 mm and numerical aperture of 0.4–0.45. For the fundamental investigation of the selectivity as a function of the laser parameters only the sample is moved using two linear axes (*x*, *y*) and one axis to move the focusing optics (*z*) to write single lines of modifications inside the glass [5]. The polarization of the laser radiation is linear and perpendicular to the writing direction to align the laser-induced nanostructures (nanoplanes, coherently continued sub-wavelength ripples) parallel to the channel to maximize the etching length. 

Since focusing inside a plane glass sample results in spherical aberrations a depth-depended compensation is included in the system. Depending on the working distance of the microscope objective used a maximum depth of 5 mm or 7 mm inside quartz glass can be processed today. The maximum area for processing is limited only by the axis system to 80 × 120 mm^2^ (scientific version of the LightFab 3D Printer with holder for four objective slides) or 200 × 200 mm^2^ (manufacturing version of the LightFab 3D Printer with vacuum chuck for wafer up to 8 in diameter) or even larger in special versions available on request.

For 3D printing of complex structures the laser focus is moved with the high dynamic 3D Microscanner inside the LightFab 3D Printer and the sample is moved in steps of up to 1 mm to modify large structures. By this step and scan strategy most of the short vectors are written with high velocity (50–200 mm/s) and small delays between individual vectors (<1 ms) compared to the large delay (50–100 ms) caused by the time necessary for acceleration of the linear axis system. Thus the modifications are written one to two orders of magnitude faster than possible without the Microscanner so that the fabrication of more complex 3D structures is feasible and contour lines can be written with smaller curvature at large velocity resulting in a larger precision. The laser parameters, the three linear axes, the three dynamic axes in the 3D Microscanner and the safety measures are controlled using the CAM software LightFabScan using the step and scan procedure to execute the scan job. Since the scan job of a complex 3D structure may consist of millions of vectors it is generated automatically by the plugin SliceLas (available from LightFab) in the CAD software Rhinoceros 3D (from Rhino3D). The 3D data (typically provided by the partners investigating an application) is sliced to layers 1–15 µm in height (parameter: slice distance) resulting in the layers of contour lines. A subset or all of the contours are selected for automatic filling with lines separated, e.g., 2–10 µm (parameter: line distance). For 3D structures with large empty regions it may be applicable to generate a sub-grid of unfilled-cubes with edge length small enough so that such glass cubes can be washed out of the final structure, e.g., 50–500 µm to minimize laser time and total strain in the glass to avoid cracks. Vectors generated are either linear lines (filling and contours of polygonized data like stl) or segments of a circle for higher precision/smaller storage requirements for the contours of e.g., NURBS data. Those contour and fill vectors are the scanjob, transferred to the CAM software LightFab Scan on the machine and executed with adjustable processing parameters. All software mentioned above is included in the LightFab 3D Printer, which is available from us. 

For experiments with the high speed microscanner and up to 100 µm channel diameter the same laser source (Satsuma) has been used [6]. For larger channel diameter produced in the same time higher laser power is necessary: The average power has to be scaled linearly to the circles’ diameter to ensure constant dose (e.g., number of pulses deposited per length) due to the larger scanning speed. The laser radiation has been amplified using a prototype slab amplifier from Fraunhofer ILT to up to 150 W average power. Furthermore a high power ps-laser (Edgewave, wavelength 1064 nm, pulse duration 10 ps, average power <80 W, repetition rate <20 MHz) has been used to produce holes in 1-mm-thick quartz glass. 

The development of the exposed 3D structures have been conducted by chemical etching in 8 mol/L KOH at 85 °C with ultrasonic excitation for 6 h (drilling in thin glass) to several days (tunnels with >10 mm length). The used heavy-duty ultrasonic bath has been tailored for the SLE process e.g., with 99 h timer, automatic heating and cooling with temperature control, automatic water refill to compensate for evaporation and is available from us. Similar equipment is surely also available from other suppliers of tailored etching/cleaning equipment.

The selectivity of the SLE-process is the ratio of the etching rates of laser-modified material and the pristine quartz glass determined experimentally. After wet-etching the developed in-volume microchannels are being investigated by transmitted light microscopy with polarization contrast, the length of the channel L is measured and subsequently the rate of selective etching r_s_ is calculated by dividing the length with the etching time. The unetched modification appears bright due to birefringence resulting from nanogratings (NG) and/or stress induced birefringence. The etched micro channel appears black with no birefringence left behind. In order to determine the selectivity S of the etching process, the ratio of the etching rates is calculated according to S = (r_s_ + r_0_)/r_0_. Where r_0_ denotes the etch rate of non-modified quartz glass which is measured to be 0.21 ± 0.015 μm/h at a temperature of 85 °C.

## 3. Results

### 3.1. Processing Parameters for 3D Printing

**Figure 1:**
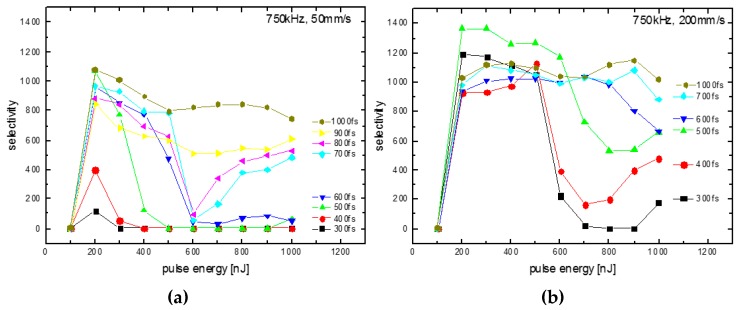
SLE selectivity vs. pulse energy with writing velocity 50 mm/s (**a**); and 200 mm/s (**b**) using a repetition rate of 750 kHz.

The selectivity (=the etching rate of modified glass divided by the etching rate of the pristine glass) depends significantly on the pulse energy and the pulse duration: at a numerical aperture of 0.4 used for these experiments, a minimum of 200 nJ is necessary to start the SLE process regardless of the pulse duration (Figure 1), thus the intensity (power density per area) of the laser radiation is not the scaling parameter for this process. A measure such as the line energy or net fluence (accumulated deposited energy per area) is not a scaling parameter of the process, since the onset does not shift using a four-times-larger writing velocity. Instead, at most pulse energies used here, a velocity of 200 mm/s results in a larger selectivity than a velocity of 50 mm/s (only 1 ps and 600 fs at 200 nJ are exceptions by a small margin). A maximum selectivity of close to 1400:1 was achieved using a velocity of 200 mm/s and a pulse duration of 500 fs (green line, Figure 1b, [5]) with the etching rate of the laser-modified quartz glass in KOH of up to 300 µm/h.

At a low writing velocity of 50 mm/s, a pulse duration of 500–1000 fs results in a maximum selectivity of 800–1100 at 200 nJ while at 400 fs the maximum selectivity is 400 and 300 fs it is about 100. At small pulse durations (300–400 fs) the range of pulse energies resulting in high selectivity (here called the processing window) is small and increases with the pulse duration. Thus for the shortest pulse duration it is very likely that higher selectivity would be observed at slightly varied pulse energy. However, for the reproducible fabrication of 3D precision glass parts a large selectivity of about 1000 is needed with reasonable tolerance to the variation of the pulse energy, pulse duration and writing velocity. Using a pulse duration of 700 fs or larger, a second processing window is observed for pulse energies >800 nJ (Figure 1a). A preliminary hypothesis to explain the existence of two processing windows is that at low energies, oxygen-deficient sections of the laser-induced nanostructures and/or resulting strain are increasing the etching rate, while at larger pulse duration micro-bubbles and/or strain due to the rapid quenching of hot (~5000 K) glass results in the increased corrodibility. A quantitative model for the laser-induced etching describing our observations is not known by the authors. At a pulse duration of 900 fs and 1000 fs, the two processing windows merge into one very large processing window. Further investigations using larger pulse durations in the ps range are promising and the subject of ongoing research, especially also for other glass materials. 

Therefore, for the 3D printing of precision glass parts, the parameters of a pulse duration of 1000 fs, a pulse energy of about 500 nJ and a writing velocity of 200 mm/s at a repetition rate of 750 kHz are optimal since all parameters may fluctuate up to 50% so that the SLE is an intrinsic stable process, which works especially well at the more productive higher writing velocity. Maintaining the high velocities even on very short vectors and sharp turns is what lets the Microscanner in the LightFab 3D Printer excel at the SLE process for 3D micro-machining. Furthermore, we conclude that for SLE, the possibility to tune the pulse duration is crucial, and it could be possible that for processing different glass materials, different pulse durations might be necessary. 

### 3.2. Up-Scaling of the Writing Speed

**Figure 2:**
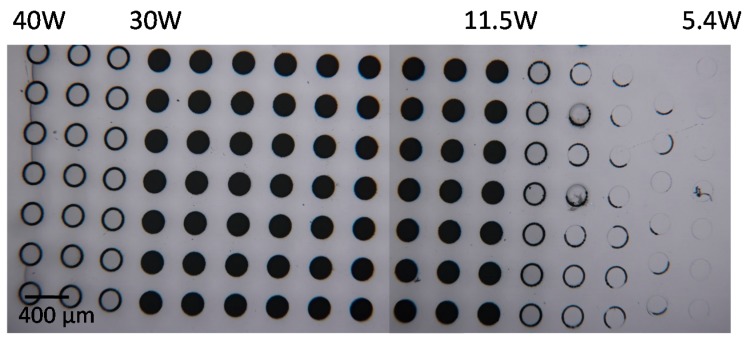
Holes 200 µm in diameter cut in 1-mm-thick quartz glass by high-speed (3 m/s) scanning a helix using 10 ps laser radiation and subsequent etching. Repetition rate: 7 MHz; exposure time per hole: 9 ms; frequency: 50 holes/s; average power varied between 5.4 W (right column) to 40 W (left column).

For the investigations of the up-scaling of the writing speed, high-power ps-laser radiation (1064 nm, 10 ps, <80 W, <20 MHz) has been focused (~2.6 µm focus diameter) using a 20× plan field objective lens (NA = 0.4, *f* = 10 mm) with the high speed microscanner module integrated into the tabletop version of the LightFab 3D Printer. In 1-mm-thick quartz glass, a helix was scanned bottom-up with a diameter of 200 µm, a velocity of 3 m/s and a thread pitch of 23 µm, resulting in a writing time of 9 ms per hole. The laser power was varied from 5.4 to 40 W in 17 steps using a repetition rate of 7 MHz. For each power the exposure was repeated 10 times with a frequency of 50 holes per second and a pitch of 400 µm. Subsequently, the sample was etched for 24 h using the conditions explained above. Using a laser power between 11.5 and 30 W (1.6–4 µJ) resulted in the successful fabrication of holes (Figure 2); using a smaller or larger laser power results in modifications not etched selectively enough so the holes stay clogged with the glass cylinders. Larger holes of up to 1 mm in diameter have been obtained by scaling the writing velocity to 13 m/s and the repetition rate to 20 MHz, and thus a laser power of 40–80 W. Thus it is concluded that the SLE process can be scaled to a large velocity (up to 13 m/s) and ps-laser radiation is suitable for the SLE process.

**Figure 3:**
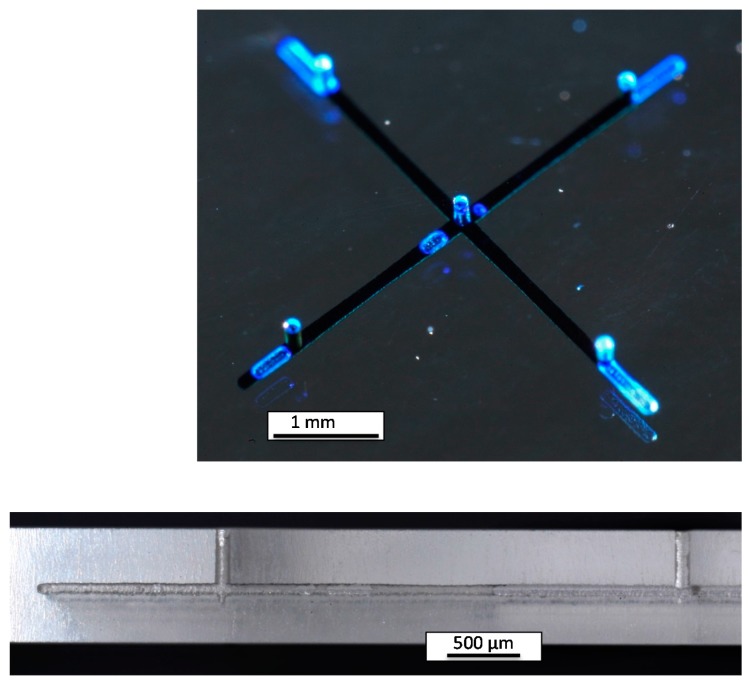
The 3D microfluidic channel exposed in less than 5 s and etched for 48 h (top) and cross-section of part of the channel (bottom) demonstrating that the SLE process can principally be scaled, making the mass-production of 3D channels feasible.

To demonstrate the feasibility to produce a 3D microchannel system at high speed, a crossing of two buried channels (length = 5 mm, width = 120 µm, height = 50 µm) and five blind holes (width = 120 µm, depth = 500 µm) was fabricated using fs-laser radiation (750 fs, 10 W, 8 MHz) and a scanning velocity of 2.66 m/s. The total exposure time was less than 5 s including the time for infeed of the axes/scanner. After etching for 48 h, the microchannel was obtained and filled with blue ink to make it visible (Figure 3); the light-blue areas are air bubbles inside the hollow channel system.

### 3.3. Up-Scaling of the Complexity of 3D Precision Quartz Glass Parts

**Figure 4:**
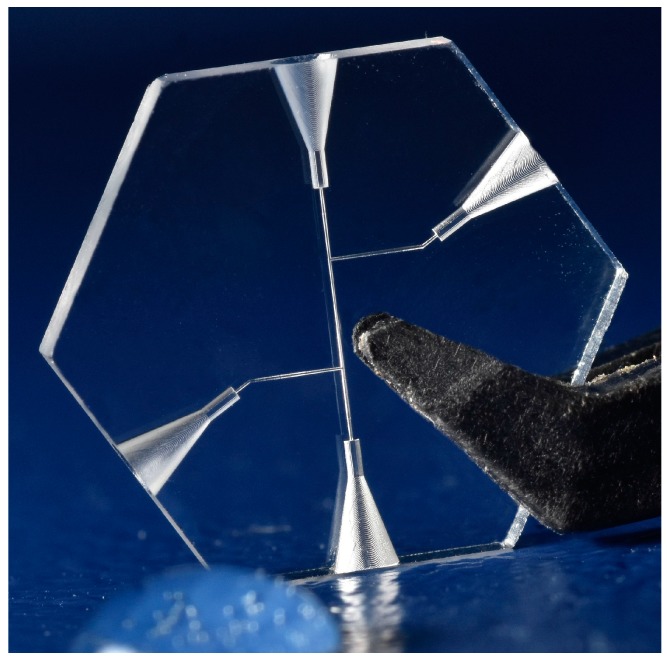
Quartz glass connector for capillary electrophoresis, diameter 15 mm, thickness 2 mm.

A connector chip for capillary electrophoresis with minimized dead volume for optimized sensitivity was produced using SLE (Figure 4). The electrophoresis application and the characterization are described elsewhere [7]; here the specific details of the generation of the scan job and the SLE processing are explained. Quartz glass 2 mm in thickness was used. Since for the SLE technique a contact of the modified volume to the glass surface is necessary, here the outline of the chip was also cut using SLE. Thus, the laser modifications for the channels are connected to the sample surfaces via the laser modifications for the trenches to cut the chip. The CAD data was sliced with a slice distance 15 µm. Only thin channels were filled with a line distance of 10 µm; thus, the female connector for the capillaries and the funnels for convenient insertion were obtained after etching by dumping the corresponding glass parts. This method reduces both the exposure time and the risk for strain-induced cracks since the laser-modified volume is minimized. Using processing parameters for 3D printing as given above, the structure was exposed layer by layer from bottom to top and developed by etching for three days as described above.

The exposure time was less than 20 min, from which the larger part was consumed for the cut-out of the chip from the wafer. However, this cut-out could also be performed by conventional mechanical cutting prior to the etching step to reduce costs in series production. Therefore, batch processing during the subtractive 3D printing is an advantage compared to in-line processing such as in additive 3D printing: since prior to the etching no microfluidic channels are present, they cannot be clogged by powder from the mechanical sawing. Only after the development in the etching process a clean environment is necessary to prevent any contamination of the microfluidic channels.

**Figure 5:**
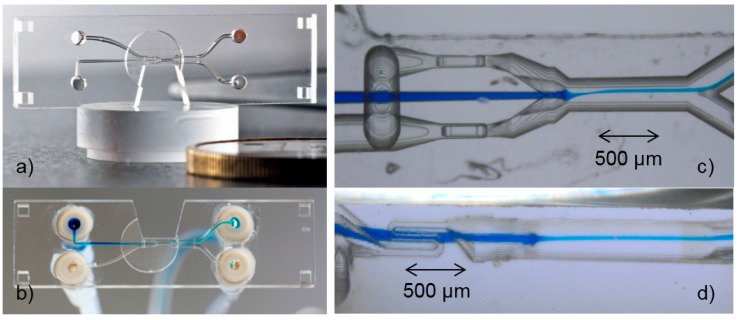
Quartz glass chip for cell sorting application, 34 mm × 12 mm × 2 mm in size: (**a**) top view of chip; (**b**) bottom view of connected chip with blue dyed analyte; (**c**) bottom view of detail; (**d**) side view of detail at the central cut-out.

A microfluidic cell-sorter chip for a quick antibiotic resistance test was produced using SLE (Figure 5). The sorting application and the characterization are described elsewhere [8]; here the specific details of the generation of the scan job and the SLE processing are explained. The outline of the chip, the four quadratic alignment holes, the trapezoid cut-out for microscopy from the side, the circular cut-out from the top for high-resolution fluorescence microscopy in 170 µm glass depth and the 3D microfluidic channels inside the chip all were produced by the SLE process in one step. 

The microfluidic channels exhibit several challenging 3D features: to increase the amount of energy absorbed in water from the switching IR-laser (the thermal change of viscosity in a channel of a small cross-section results in an increased flow on one side enabling the switching, see detailed explanation provided in [8]), the radiation must pass three times through the same channel. Thus, two channels with a small diameter must meander vertically in the direction of the laser beam (Figure 5d). Furthermore, the channel for the analyte must be a precise injection nozzle between the two sheath flow channels to enable 3D hydrodynamic focusing. Since the pristine quartz glass is also etched with a rate of about 0.2 µm/h, it was necessary to reduce the amount of over-etching at those three high-precision 3D features as much as possible by designing the distance between them and the entrance of the channel all equally. In that way the etching time at the high-precision features is minimized and so is the over-etching. 

The second challenge in producing this structure was to avoid the formation of cracks during the etching process: due to the 3D nature of the channels and the high-precision features, it is necessary to fill the channel completely with laser-modified lines, and thus quite some stress is deposited in the glass until the channels are etched out. Since the glass is only 2 mm in thickness while the channels are more than 10 mm long between the four entrances at the top surface, the cut-outs from the side and the top are completed earlier. Thus, the stress field inside the glass is increased while the mechanical stability of the glass structure is reduced during the time when the cut-outs are completed but not the channels. Therefore, the strain field and the risk for cracking were reduced by avoiding sharp corners and using rounded features in the critical central parts of the design. Further iterations and design changes were related to the switching performance of the chip and are out of the scope of this paper.

The final design was sliced with a 15 µm slice distance and the contours in the microfluidic channels were filled with a line distance of 9–12 µm. Using processing parameters for 3D printing as given above, the structure was exposed layer by layer from bottom to top (the exposure time was less than 20 min) and developed by etching for four days as described above.

**Figure 6:**
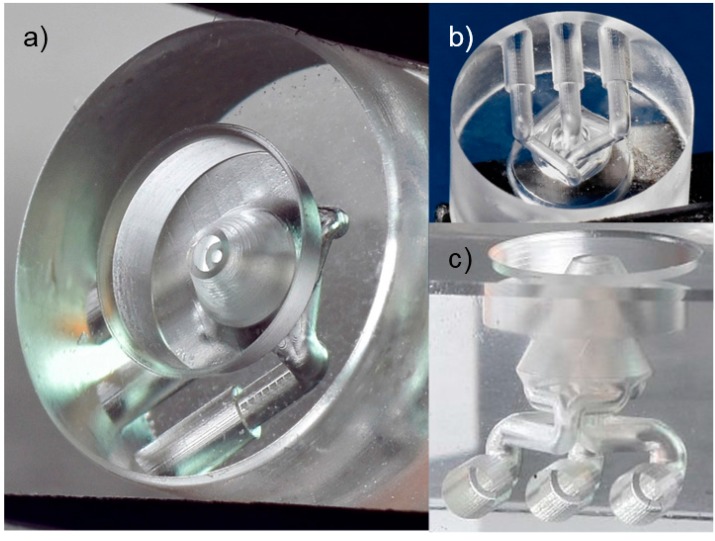
Nested nozzle in quartz glass for biological application (diameter: 10 mm, height: 7 mm): (**a**) top view; (**b**) bottom view; (**c**) side view of nozzle produced in glass cube for clearer view.

A nested 3D nozzle for the cell breeding application was produced in quartz glass using the SLE process (Figure 6). The application is currently under investigation as well as further design modifications, and might be published later by the University of Genova. The challenge in producing this design was that precise circular shapes are needed both in the *x*-*y* plane (openings of the nozzles) and in the *z*-*y* plane perpendicular to it. While a circular shape in the *x*-*y* plane is easily achieved by writing circles with the scanner, a circular shape in the perpendicular plane is approximated by steps due to the slicing (layer-by-layer writing). Choosing a layer height (parameter slice distance) of 1 µm or smaller would result in a long fabrication time and also a large accumulation of stress increasing the risk for cracking. Thus, adaptive slicing was used to produce this structure: contour surfaces with a small inclination angle towards the *z*-axis (parallel to the laser beam propagation) were sliced with 15 µm while the slice distance was reduced with a larger inclination angle down to 1 µm in steps of 1 µm. Only selected contour layers with a height separation of 15 µm were chosen in the software to be filled with a line distance of 10 µm. 

In this way a fast exposure (<10 min, parameters given above) and a large precision (deviation from circle <2 µm) are obtained simultaneously.

## 4. Discussion

The highest selectivity, which is the ratio of the etching rates of the laser-modified quartz glass (up to 300 µm/h) and the unmodified quartz glass (0.21 µm/h), of up to 1400 was obtained for the SLE of quartz glass and etching in hot KOH. Surprisingly, 1 ps laser radiation results in larger selectivity and longer processing windows than shorter femtosecond laser radiation. We conclude that the pulse duration is an important parameter during the SLE process development and probably also for other glasses and crystals, and that an ultrafast laser with tunable pulse duration is necessary for future investigations. In further ongoing investigations we will expand the pulse duration in the few-ps range especially. 

Using a repetition rate of 750 kHz, the larger writing velocity of 200 mm/s results in a larger selectivity, and thus the processing precision is larger than obtained using 50 mm/s writing velocity. It is a unique observation that faster laser writing results in larger precision. Furthermore, the parameter window, which is the width of the range of pulse energies suitable for SLE, is increased with larger velocities. Thus, the SLE process is not only more precise but also more robust when using larger writing velocities. Finally, it is of practical benefit to complete a job in shorter time by faster writing. 

High-speed SLE was used to drill 200 µm holes in 1-mm-thick quartz glass and the scaling of the SLE process was demonstrated with a 3 m/s writing velocity and a 10 ps laser radiation in only 9 ms (50 holes per second including the infeed time). The possibility to scale the SLE process for the mass production of 3D parts was demonstrated by a 3D microchannel exposed in 5 s. Thus, millions of 3D precision glass parts can be produced in the future using a single mass production machine including one of our high-speed Microscanners tailored to the customers’ design. We have also demonstrated that ps-laser radiation is suitable for the SLE process using a high writing speed. 

Up-scaling of the complexity of 3D precision parts was achieved by a flexible and automatic CAD/CAM software chain including adaptive slicing and variable filling strategies. The further increase of the precision of the 3D glass parts was achieved using curved vectors automatically generated from 3D CAD data avoiding the polygonization errors from, e.g., STL files. Next to the 3D design, the outline of the chip including precise structures for alignment was also produced in the same processing step, reducing the need for post-processing or further precision alignments. After etching and cleaning, such 3D precision glass parts are ready to use. 

Some examples for design optimizations during the construction process for subtractive 3D printing have been explained and the new freedom in 3D printing has been demonstrated. It is important to learn that in subtractive 3D printing, no support structures are needed and any angles are possible, and thus the design freedom is significantly larger than in additive 3D printing. Furthermore, the precision is larger (it can be less than 1 µm) and the material properties are unchanged from the suppliers’ data sheets, since all the laser-modified material is removed after etching and the resulting structure is free of strain.

One disadvantage of the subtractive 3D printing process SLE in comparison to an additive process such as selective laser melting (SLM) is the missing in-line capability: after laser processing, the structure must be developed in a separate etching batch process. Thus, for any post-treatment the structure must be aligned again. However, post-treatments are only necessary for optical polishing needs since the surface finish after SLE is like a fine-grinded surface (peak-to-valley roughness Rz < 1 µm) and the alignment needs for subsequent laser polishing are minor [9]. Furthermore, we have demonstrated above that the missing in-line capability can be a clear advantage, especially for microfluidic applications: since the channels are present only after the etching, various post-processing methods such as mechanical sawing, grinding or milling can also be used after laser treatment without clogging the microchannels with dust particles. Thus, only after the final development of the structure in the wet-chemical etching, the part must be handled in clean room conditions. 

We hope that these results will stimulate further investigations in various disciplines to enable a wide application of subtractive 3D printing using the SLE technology in the future.
